# New genetic mutations in a Chinese child with Ehlers-Danlos syndrome-like spondyloepimetaphyseal dysplasia: A case report

**DOI:** 10.3389/fped.2022.1073748

**Published:** 2022-12-21

**Authors:** Shu Han, Xuan Xu, Jie Wen, Jianzhou Wang, Sheng Xiao, Li Pan, Jiang Wang

**Affiliations:** ^1^ Children's Medical Center, Hunan Provincial People's Hospital, The First Affiliated Hospital of Hunan Normal University, Changsha, China; ^2^ Department of Pediatric Orthopedics, Hunan Provincial People's Hospital, The First Affiliated Hospital of Hunan Normal University, Changsha, China; ^3^ Department of Pediatric General Surgery, Hunan Provincial People's Hospital, The First Affiliated Hospital of Hunan Normal University, Changsha, China

**Keywords:** Ehlers-Danlos syndrome, mutation, genetics, sequence analysis, case report

## Abstract

**Background:**

Ehlers-Danlos syndrome (EDS) spinal deformity type 2 has clinical features similar to those of spondyloepimetaphyseal dysplasia with joint laxity, type 1 (SEMDJL1). They have similar clinical manifestations and a similar genetic basis, both of which can be caused by mutations in the *B3GALT6* gene. Hence, genetic screening and careful clinical examination are key to the differential diagnosis of these two diseases.

**Case presentation:**

A 4-month-old boy was admitted to our hospital in order to find the causes of developmental delay. The clinical examination revealed that the child was delayed, with an excessive range of motion of joints, patent foramen ovale, and was accompanied by skin aging; the child was suspected to have EDS. However, unlike EDS, the child had normal muscle tension, and at the same time had a spinal deformity, mild kyphosis, widened right hip joint space, as well as a special face, joint laxity, and slender fingers, which were typical characteristics of SEMDJL1. A gene analysis showed two suspicious mutations in the *B3GALT6* gene: c.808G > A(p.(G270S)) and c.942G > C(p.(W314C)), which were verified to be compound heterozygous mutations by analyzing genes in his parents. This mutation was not included in the HGMD, ClinVar, and other mutation databases, and thus was a newly discovered mutation.

**Conclusion:**

Using the clinical and genetic analyses, this study reported a Chinese case with EDS-like SEMDJL1 for the first time. Two pathogenic mutations were discovered in the *B3GALT6* gene: c.808G > A(p.(G270S)) and c.942G > C(p.(W314C)).

## Background

Ehlers-Danlos syndrome (EDS) is known as dermatorrhexis with hyperelastia cutis accompanied by cutis laxa and joint laxity, which is a connective tissue disease and manifests as joint pain, swelling, and instability, as well as spinal deformity if it involves the bone and joint systems. EDS spinal deformity type 2 manifests as an aging appearance, motor and cognitive retardation, short stature, craniofacial disproportion, extensive bone loss, defects in wound healing, an excessive range of motion of the joints, low muscle tension, and loose but elastic skin (1). It has a variety of genetic patterns, including autosomal dominant, autosomal recessive, or X-linked recessive inheritance.

Spondyloepimetaphyseal dysplasias (SEMD) usually manifest as vertebral dysplasia (2) and are often accompanied by other clinical features. Spondyloepimetaphyseal dysplasia type I (spondyloepimetaphyseal dysplasia with joint laxity, type 1, with or without fractures, SEMDJL1; OMIM 271640) is a common subtype of SEMD usually accompanied by joint laxity; some SEMDs may be associated with fracture performances. The main clinical manifestations are vertebral dislocation, kyphoscoliosis, excessive ligament laxity, joint laxity, and dislocation. Some patients have special faces, such as an elliptical face, flat-center face, long philtrum, exophthalmos, or blue sclera (3). This disease is associated with autosomal recessive inheritance. It is caused by a pathogenic mutation in the *B3GALT6* gene (OMIM 615291) (4–6). The *B3GALT6* gene encodes β-1,3-galactosyltransferase and plays a role in the synthesis of heparin sulfate and chondroitin sulfate. Its mutation may also cause EDS spinal deformity type 2.

The clinical features of EDS spinal deformity type 2 are similar to those of SEMDJL1. They have similar clinical manifestations and genetic basis, both of which can be caused by mutations in the *B3GALT6* gene. Hence, genetic screening and careful clinical examination are the keys to the differential diagnosis of these two diseases. This study reported a Chinese child, in which characteristics of EDS were found through careful examination and comparison with other cases reported previously, such as motor retardation, aging appearance, slender fingers, an excessive extension of joints, cardiovascular atrial septal defect, and patent foramen ovale. At the same time, the patient also appeared to have typical features of SEMDJL1, such as a special face, spinal deformity, slight kyphosis, and widened right hip joint space. To identify whether it was a metabolic disease and its underlying causes, the whole exome was detected by high-throughput sequencing, and two suspicious mutations were found in the *B3GALT6* gene. Based on the mutations in his parents, these two mutations constituted a compound heterozygous mutation, which was pathogenic. Therefore, the child was diagnosed with EDS-like SEMDJL1 based on the aforementioned clinical features and gene sequencing results. The follow-up continued to track the development of his spinal deformity and facial features. These results of the study can be used as a reference for identifying such diseases in the Chinese population.

## Case presentation

A 4-month-old boy who could not look up was admitted to our hospital in order to find the causes of the developmental delay. His birth weight was 2.5 kg, without deformity and bleeding. He was diagnosed with infectious pneumonia, myocardial damage, and hypokalemia in his neonatal period, which improved after receiving CPAP-assisted ventilation, amoxicillin clavulanate potassium, and other symptomatic support treatments, and was discharged after the symptoms improved. There was no abnormality in the paternal familial genetic history, while the mother was adopted and her family history was unknown.

**Figure 1:**
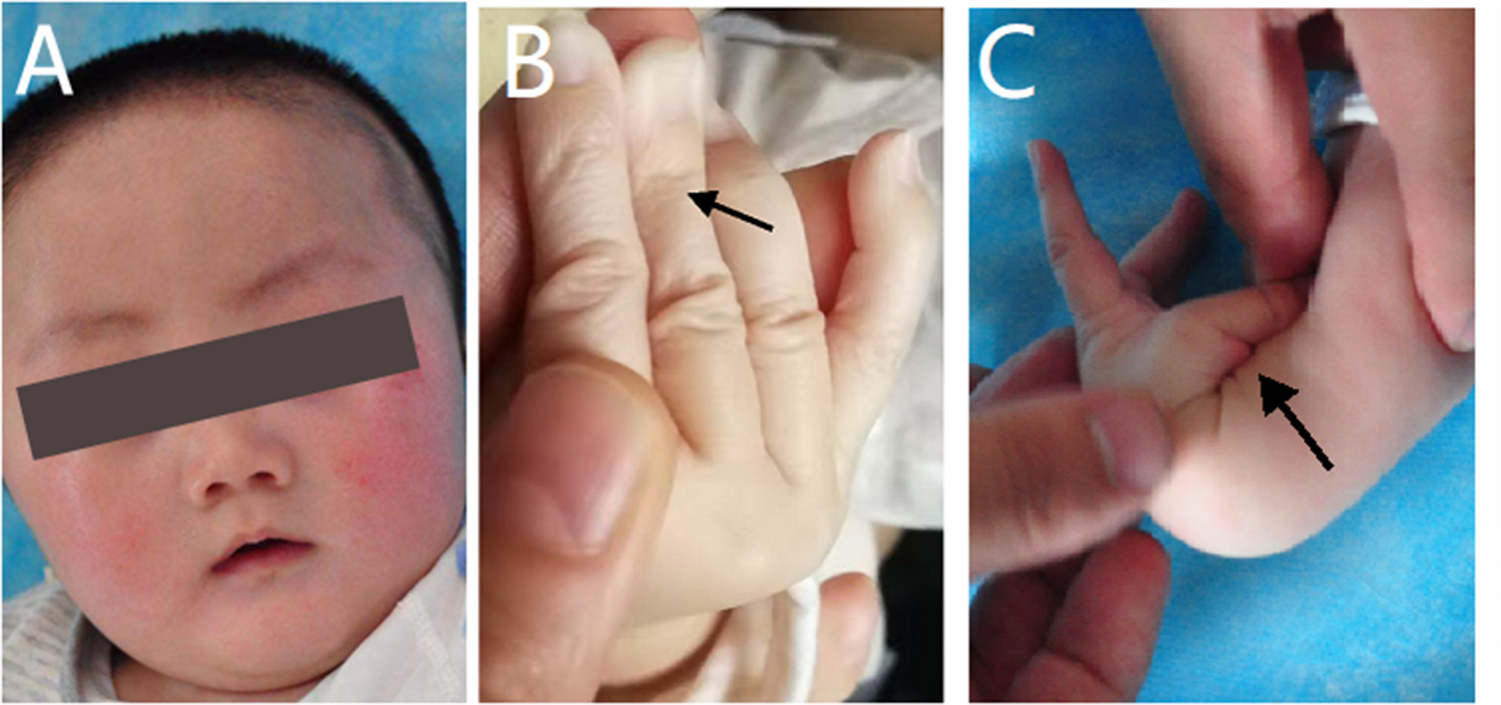
Appearance photos of the patient. (**A**) The patient had a special face like thickened inner canthus and rough skin in the face. (**B**) The patient's finger was slender and the skin was aging. (**C**) The patient appeared increased range of motion of joints in the hands, with a dorsal extension of about 110°.

The physical examination at admission showed motor retardation, short stature, inability to look up at the age of 4 months, a special face with a thickened inner canthus, rough skin on the face, and a slightly thicker tongue coating (Figure 1A). These clinical features were in accordance with the characteristics of SEMDJL1. However, the possibility of metabolic diseases could not be ruled out. At the same time, the patient's fingers were slender and his skin was aging (Figure 1B). The aging skin and cutis laxa were consistent with the characteristics of EDS. In addition, he also appeared to have ligament laxity and an increased range of motion of the joints in his hands, with a dorsal extension of approximately 110° (Figure 1C).

**Figure 2:**
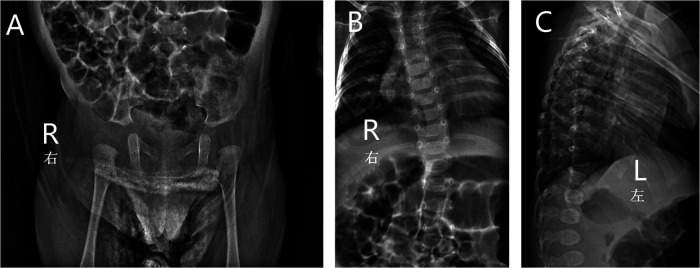
X-ray of the patient showed widened right hip joint space (**A**), spinal deformity, dislocation (**B**) and slight kyphosis (**C**).

Ultrasonography showed a cardiovascular atrial septal defect, patent foramen ovale, and left hydrocele testis. An x-ray examination found spinal deformity, dislocation, slight kyphosis, and a widened right hip joint space, which were typical manifestations of skeletal dysplasia and similar to the excessive joint extension as mentioned above (Figure 2). Other examinations were performed: laboratory tests revealed that the child had a low concentration of vitamin D, which may have led to insufficient bone calcification and resulted in skeletal dysplasia, and is proof for metabolic disease. The child had normal hearing and karyotype.

Combined with the clinical features and auxiliary examination of the child, it was not possible to confirm the diagnosis of EDS or SEMDJL1, both of which were caused by the mutation in the *B3GALT6* gene and had clinically overlapping features; therefore, a further molecular genetic analysis was performed to allow for confirmation of the exact diagnosis, guide management, and give information on inheritance pattern, risk of recurrence, and prognosis.

**Figure 3:**
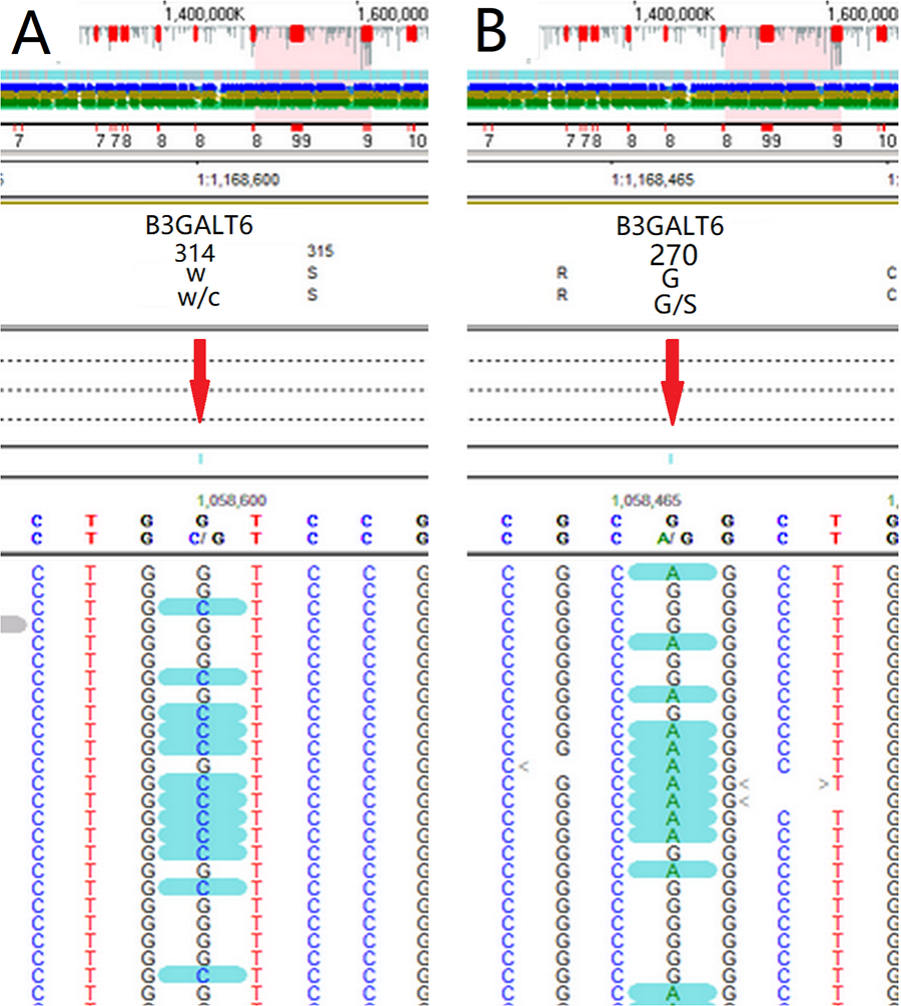
Whole exon gene sequencing showed that there were two suspicious mutation sites in the *B3GALT6* gene: c.942G>C(p.(W314C)) (**A**) and c.808G>A(p.(G270S)) (**B**).

Whole exon gene sequencing showed that there were two heterozygous mutation sites in the *B3GALT6* gene: c.942G>C(p.(W314C)) (Figure 3A) and c.808G>A(p.(G270S)) (Figure 3B). The mutations were further analyzed, and the results revealed that both mutations were missense mutations, which were defined as variant of uncertain significance in ACMG classification (7).

**Figure 4:**
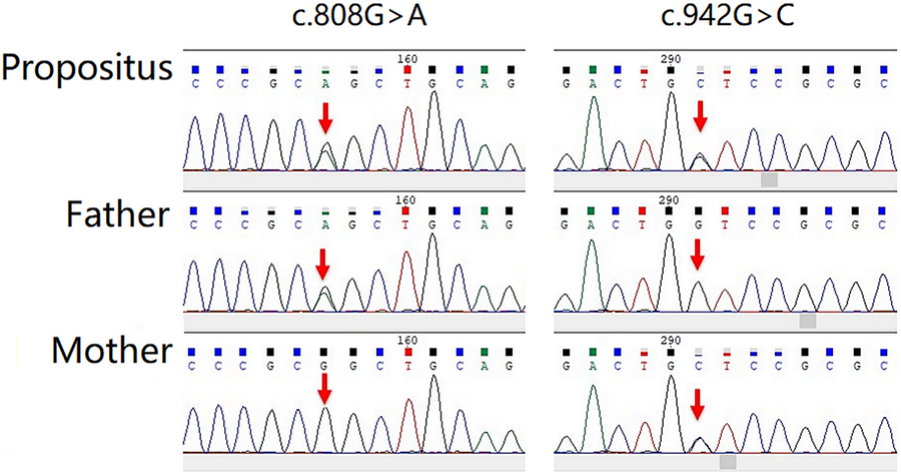
The gene sequencing gold standard sanger method was used to verify the mutation, and it was found that the child showed mutation at the loci of the suspected mutation.

The gene sequencing gold-standard Sanger method was used to verify the mutation, and it was found that the child showed a mutation at the loci of the suspected mutation. Among them, in the c.808G > A, the father had a mutant type, and the mother had a wild type; in the c.942G > C, the father had a wild type, and the mother had a mutant type (Figure 4).

From the above results, the two mutations in the child were derived from his parents, which constituted a compound heterozygous mutation. The parents were carriers without clinical manifestations. Meanwhile, the child was a compound heterozygote, and the clinical manifestation was consistent with autosomal dominant inheritance, which was the genetic pattern of EDS and SEMDJL1. Therefore, EDS-like spondyloepimetaphyseal dysplasia with joint laxity, type 1 was confirmed. For the two mutations detected, there was a lack of mutation frequency statistics in 1KGP (8), dbSNP (9), HapMap (10), and ExAC (11), and they were not recorded in HGMD (12), CliVar (13), or other mutation databases. They were newly discovered suspected mutations. A human position hip brace was given to maintain a good hip joint reduction. After treatment, the hip joint was maintained in a good position.

## Discussion

This study reported on a Chinese child with skeletal dysplasia. Clinical examination revealed that the child was developmentally delayed, unable to look up at the age of 4 months, with an excessively low level of vitamin D, and was suspected to have metabolic disease at admission. Nonetheless, the possibility of metabolic diseases was ruled out by examinations such as blood routine, urine routine, stool routine, liver function, renal function, myocardial enzymes, electrolytes, blood glucose, C-reactive protein, abdominal B-ultrasound, chest X-ray, echocardiography, respiratory virus infection, blood ammonia, thyroid function, cortisol, ceruloplasmin, B-ultrasound of the scrotum and testicles, and plain and enhanced MRI of the head. At the same time, he was found to have an excessive range of motion of his joints, patent foramen ovale, and skin aging, and was considered to have EDS. However, the child also experienced spinal deformity, mild kyphosis, and a widened right hip joint space, which were typical characteristics of SEMDJL1. Further molecular genetic analysis showed that there were two suspicious mutation sites in the *B3GALT6* gene, which could cause SEMDJL1 and EDS. Unlike EDS, the child had normal muscle tension, normal hearing, and an obvious special appearance, and thus was diagnosed as EDS-like SEMDJL1. In addition, the child had manifestations of hydrocele testis, which were not found to be related to any genetic diseases and might be only the sporadic manifestation of the disease.

SEMD, which manifests as skeletal dysplasia, has many subtypes and a complex molecular mechanism. It is generally believed that SEMDJL1 is caused by a mutation in the *B3GALT6* gene. This gene is located at 1p36, which encodes the β-1,3-galactosyltransferase and plays a role in the synthesis of heparin sulfate and chondroitin sulfate. It is usually located in the Golgi apparatus and mainly catalyzes the UDP-galactose to make it transfer and bind to the substrate (4). SEMDJL1 manifests as symptoms of typical skeletal dysplasia, such as vertebral dislocation and spinal kyphosis, but also presents as non-axial skeletal deformities (14), such as radioulnar dislocation, hip dysplasia, and finger ulnar deviation. SEMDJL1 is usually also accompanied by joint laxity, excessive extension, thoracolumbar scoliosis, hip subluxation, and slender fingers (15). The most common abnormality of the spine is kyphoscoliosis. For the patient in the present study, the x-ray showed only slight kyphosis, which might be related to the age of the child. Therefore, concerns about kyphosis are particularly important in future follow-ups. Of course, some of the cases with this disease have a special face, such as an oval face, flat midface, prominent eyes with blue sclerae, and a long philtrum, or cognitive disorders rather than skeletal dysplasia (4, 5). The child in this study also had a special face, and his cognitive impairment was temporarily undetectable due to his young age, which should also be an important part of future follow-ups.

*B3GALT6* mutations can also cause EDS. Deletion in this gene may lead to defects in the initiation of glycosaminoglycan synthesis, thereby resulting in pleiotropic EDS-like connective tissue disorders. Some clinical manifestations of EDS are similar to those of SEMDJL1, which is probably due to some similarities in their pathogenesis. In addition, patients of different races may present with different clinical features, even with the same mutation mechanism. Studies have found that in sporadic cases in South Africa, cases with the *B3GALT6* gene mutation are often of the SEMDJL1 type, while the *B3GALT6* gene mutation often leads to EDS in the Japanese population (16). In the present study, this gene mutation led to typical manifestations of EDS-like SEMDJL1 in the Chinese population. The patient was diagnosed as having SEMDJL1 based on two reasons: first, the molecular mechanism and genetic pattern of SEMDJL1 were in line with his clinical features; and second, the skeletal dysplasia exhibited is the major feature in the patient. The facial appearance of EDS may be different from that of SEMDJL1. Usually, most patients with SEMDJL1 show abnormal appearances, while some may have the normal appearance (17). In this study, the child had a special appearance, a thickened inner canthus, rough skin on the face, and a slightly thick tongue coating, which may be different from the features of EDS (18). EDS itself is complex, and its differential diagnosis is particularly difficult. The differential diagnosis between EDS and SEMDJL1 was also a challenge for clinicians. An accurate diagnosis could only be achieved by distinguishing their clinical features and molecular mechanisms. SEMDJL1 caused by mutation of the *B3GALT6* gene was not found to be associated with hearing loss. In addition, individuals with EDS may have hearing loss (19). The child in this study had normal hearing and a *B3GALT6* gene mutation, which was one of the pieces of evidence for the diagnosis of SEMDJL1.

In the present study, a vitamin D deficiency was found in the child, which might be one of the factors leading to skeletal dysplasia. Two of the outcomes might be an excessive range of motion of the joints and aging skin, which are similar to the typical characteristics of EDS. As a typical connective tissue disorder, many factors originate from the genetic basis of EDS. In a newly discovered class of EDS, a common feature has been found, i.e., a mutation in the *CHST14* gene (20–23). Studies have found that vascular abnormalities may occur in the new EDS (24), which is similar to the manifestations in the patient in the present study. Ultrasonography showed a patent foramen ovale and atrioventricular defect, and we tested the patient for *CHST14*. There was no mutation site in the *CHST14* gene of the patient, and the significance of the *CHST14* gene remains to be investigated in the definite diagnosis of EDS. Another typical feature of EDS is its aging appearance. The patient in this study showed similar characteristics; his skin was aging but to a mild degree.

## Conclusion

The present study reported a Chinese child with skeletal dysplasia that was diagnosed as EDS-like SEMDJL1, which revealed two suspicious mutations in the *B3GALT6* gene to form a compound heterozygous mutation: c.808G > A(p.(G270S)) and c.942G > C(p.(W314C)). This mutation was not included in the HGMD, ClinVar, and other mutation databases, and was a newly discovered mutation. This study provided the experimental basis for the differential diagnosis between skeletal dysplasia and connective tissue abnormalities in the Chinese population.

## Data Availability

The original contributions presented in the study are included in the article/Supplementary Material, further inquiries can be directed to the corresponding author.
